# Limiting *trans* Fats in Foods: Use of Partially Hydrogenated Vegetable Oils in Prepacked Foods in Slovenia

**DOI:** 10.3390/nu10030355

**Published:** 2018-03-15

**Authors:** Nina Zupanič, Maša Hribar, Urška Pivk Kupirovič, Anita Kušar, Katja Žmitek, Igor Pravst

**Affiliations:** 1 Nutrition Institute, Tržaška cesta 40, SI-1000 Ljubljana, Slovenia; nina.zupanic@nutris.org (N.Z.); masa.hribar@nutris.org (M.H.); urska.pivk.kupirovic@nutris.org (U.P.K.); anita.kusar@nutris.org (A.K.); katja.zmitek@vist.si (K.Z.); 2 VIST—Higher School of Applied Sciences, Gerbičeva cesta 51A, SI-1000 Ljubljana, Slovenia

**Keywords:** *trans*-fatty acids, partially hydrogenated oils, coronary heart disease, food policy, Slovenia

## Abstract

Consumption of industrially produced *trans*-fatty acids (TFAs) is a well-established health risk factor that correlates with the increased risk of developing cardiovascular disease. The recommended TFA intake is as low as possible, within the context of a nutritionally adequate diet. Different countries have introduced different measures to minimize the exposure of their population to TFAs. Previous data have shown that TFA content has significantly decreased in Western European countries, while this was not the case in many Central-Eastern European countries, including Slovenia. In the absence of regulatory requirements, a number of awareness campaigns were launched in Slovenia since 2015, with the common goal of lowering the use of partially hydrogenated oils (PHO), which are considered a major source of TFAs. To determine if this goal had been reached, we performed an assessment of the exposure of the population to prepacked foods containing PHOs in years 2015 and 2017. Altogether, data on the composition of 22,629 prepacked foods was collected from food labels, using a specifically developed smartphone application. Furthermore, the food categories with the most frequent use of PHOs were identified. The proportion of PHO-containing products was determined for each specific food category, and adjusted with the market share data. The results showed that in 2015, vegetable cream substitutes, soups, and biscuits were the categories with the highest penetration of declared PHO content. In 2017, the proportion of products with PHO decreased considerably. In vegetable cream substitutes the percentage of PHO containing items dropped from 30 down to 4%, in soups it decreased from 21 to 5%, in biscuits from 17 to 8%, and in crisps and snacks from 10 to 4%. However, PHO content remained notable among cakes, muffins, pastries, and biscuits. We can conclude that the voluntary guidelines and regular public communication of the risks related to the TFA consumption has had a considerable effect on the food supply, but did not result in sufficient removal of PHOs from foods.

## 1. Introduction

*Trans*-fatty acids (TFAs) are unsaturated fatty acids with at least one double bond in the *trans* configuration [1]. Certain TFAs are naturally produced by the bacteria in the rumen and are therefore found in the meat, milk and dairy products of ruminants [2]. However, their contribution to overall TFA consumption is minimal [3]. On the other hand, partially hydrogenated vegetable oils (PHOs) are considered to be the major source of industrial TFAs in people’s diets. The process of industrial hydrogenation of vegetable oils results in several desirable properties of the product: it increases the stability and solidity of the oil, subsequently increasing the shelf life of the final product while decreasing the need for its refrigeration. In addition, PHOs tend to be cheaper than animal fats and were, for a long time, thought to be a healthier alternative to saturated fats of animal origin, such as butter and lard [1].

As early as the 1990s, the first scientific evidence appeared correlating TFAs with raised plasma low-density lipoprotein (LDL) and decreased high-density lipoprotein (HDL) levels [4,5]. Results from metabolic studies were later substantiated by epidemiological studies, which showed a positive correlation between TFA intake and risk for cardiovascular disease (CVD) [6,7,8,9,10]. The United States Centre for Disease and Prevention estimated that industrial TFA consumption accounts for as many as 20,000 cases of CVD and 7000 deaths per year in the US alone [11]. The World Health Organisation [12], as well as the European Food Safety Authority [13], have recommended that TFA intake should be as low as possible within the context of a nutritionally adequate diet. This is reflected in the reformulation of many processed food products; responsible food manufacturers began to reformulate their food products and stopped using PHOs, while policymakers started to regulate this area.

Different measures were accepted in different jurisdictions to minimize the content of industrial TFAs in foods. One of the fastest and most radical responses happened in Denmark only shortly after the paper lead by Willett et al. [5] was published in March 1993. The Danish margarine industry had been willing to cooperate with Danish health professionals and had gradually eliminated industrially produced TFAs from their products even before the legislative ban was enacted in 2003 [14]. In Europe, a similar approach was later undertaken by Switzerland, Austria, Iceland, Hungary, Norway, and Latvia [15]. Another possible way to tackle the problem is to enact mandatory labelling of either TFAs or PHOs. In 2005, such labelling was introduced in prepacked foods in Canada, while the United States (US) followed shortly after [14]. Mandatory labelling of the presence of PHOs is currently in use in the European Union (including Slovenia), but the efficacy of such approach remains questionable [16,17]. In this way, consumers need to firstly identify PHOs within the list of ingredients on the food label, and subsequently recognize such ingredient as potentially hazardous due to its TFA content. Recently, US has introduced a more efficient strategy [18], where PHOs can no longer be considered as “generally recognized as safe” (GRAS) for use in human food, and will thus be banned by June 2018.

CVDs are still the most frequent cause of death globally. Every year, more than 4 million people die due to CVD in Europe alone, accounting for more than 40% of all deaths in Europe [19]. In Slovenia, CVDs were the cause of 38% of all deaths and are associated with 10% of total health care costs. Occurrence of CVDs in Slovenia is the highest amongst members of the lowest socio-economical class, indicating the importance of lifestyle-related risk factors, as well as healthcare inequalities within Slovenian society [20]. One of the important risk factors for CVD is consumption of industrially produced TFAs, which are still present in Slovenian prepacked and non-prepacked foods. Even though in Slovenia all prepacked foods that contain PHOs have to clearly indicate this on the list of ingredients, it is very difficult for consumers to perceive this as a health hazard [21]. This became very evident in 2013, when Stender and colleagues examined the TFA content in prepacked biscuits, cakes, and wafers from 20 European capitals, including Ljubljana, Slovenia. Their findings were worrisome; a number of foods bought in Slovenia contained industrial TFAs, some with concentrations of up to 9 g per 100 g of product [22]. Even more concerning results were reported in their subsequent study, which showed that in Slovenia, the availability of biscuits with added PHOs had increased by almost 300% from 2012 to 2015, as did the TFA levels [23]. A similar pattern was also observed in several other Central Eastern Europe (CEE) countries.

**Figure 1:**
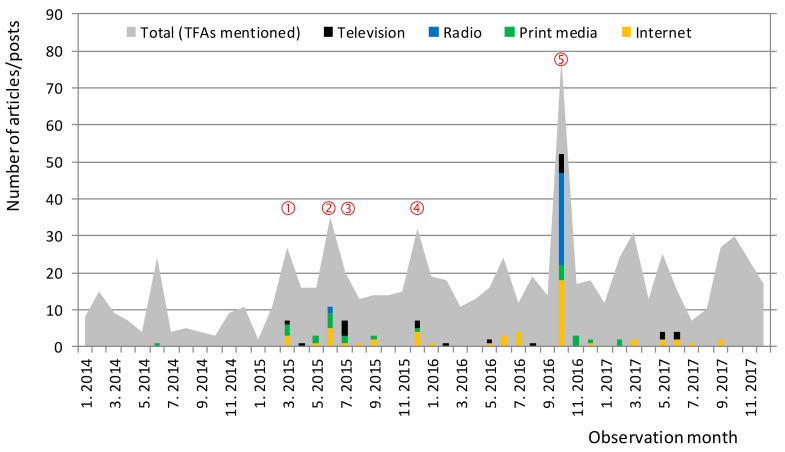
Press coverage of *trans*-fatty acids issue (TFAs) (Slovenia; 2014–2017). Notes: Figure 1 was constructed using the press coverage data purchased from Kliping media agency (Slovenia), which collects full texts of publications from all important media channels in Slovenia (covering major television and radio stations (transcripts), print media, and internet portals). Number of publications that mention TFAs is provided in grey; publications that focus on TFAs are represented as bars (black: television; blue: radio; green: print media; yellow: internet). Press coverage peaks correspond with: ① Scientific conference on the TFAs in foods (March 2015; press conference); ② News about the decision in the USA to remove PHOs from the GRAS list (June 2015; [18]); ③ Edition of “Tarča” show “Trans fats: hidden, dangerous, legal” was broadcasted on Slovenian national television, exposing specific food brands high in TFAs in the Slovenian food supply (July 2015; [25]); ④ Publication of the report from the European commission regarding TFAs in foods and in the overall diet of the Union population (December 2015 [26]; press release published); ⑤ Presentation of the “Trans Fats in Foods in Slovenia”(TFFS) national research project in the media (October 2016, press conference covered by all major national mass media).

Considering these facts, it became a major national public health priority to lower levels of TFAs in foods and their dietary intake [24]. Several awareness campaigns were launched in 2015 and 2016, targeting media, consumers, and food business operators. Media coverage of the issues related to TFAs increased, building pressure on food manufacturers to remove PHOs from the foods (Figure 1). Additionally, a research project “Trans Fats in Foods in Slovenia” (TFFS) was funded with the objective of investigating the exposure of the population to TFAs and informing policymakers in making evidence-based policy decisions. The objectives of the first part of the project and this particular study were to supplement a national food composition database with information on the presence of PHOs in prepacked foods, to identify possible contributors to TFA consumption, and to evaluate the changes in the use of PHOs between 2015 and 2017. The reported results provide necessary evidence for more efficient interventions in future, not only at the EU level, but also globally.

## 2. Experimental Section

### 2.1. Data Collection

Two consecutive cross-sectional studies were conducted, one in the beginning of 2015 and the following one in the beginning of 2017. Each year, data on the composition of prepacked foods in the Slovenian food supply was collected from the two major grocery chains with the largest nationwide shop networks (Spar, Mercator). With the agreement with the retailers, all prepacked products with a unique European/International Article Number (EAN) barcode were systematically photographed and recorded in an online Composition and Labelling Information System (CLAS) database [27]. To accelerate the database’s formation and to avoid duplicate entries, the data collection was supported by a specially developed computer application, which enables the digital recognition of EAN codes. The information collected included the name of the product, list of ingredients, nutritional values, packaging volume, price, and EAN barcode. However, only presence of PHOs is reported for the purposes of this study. The CLAS database was further complemented with country-wide, 12-month sales data obtained from both retailers. These data present the number of items sold in a 12-month period prior to the start of the data collection. The sales data were received in the universal form, including the European/International Article Number (EAN barcode), description of the product, number of products sold per year, and the quantity of food (kg/L) per packaging. The sales data provided by different retailers were combined to obtain the overall national yearly sales data for each product. The matching was performed using EAN barcodes. For the purpose of this study, a food categorisation system developed within the Global Food Monitoring Group by Dunford et al. was utilised [28]. One deviation that was made from the original categorisation system was the separation of creams and vegetable cream substitutes into two separate categories, due to the observed common use of PHOs in vegetable cream substitutes (e.g., vegetable oil-based whipping creams). After the exclusion of food categories without any PHO-containing items, analyses were conducted on the following 26 food categories: Biscuits; bread; breakfast cereals; butter and margarine; bakes, muffins and pastries; cereal bars; cheese; chilled fish; chocolate and sweets; coffee and tea; cooking oils; cream; crisps and snacks; desserts; honey and syrups; ice cream and edible ices; noodles; pasta; pizza; pre-prepared salads and sandwiches; processed meat and derivatives; ready meals; sauces; soup; spreads; and vegetable cream substitutes. The sample included all foods available in the selected grocery stores at the time of sampling for which sales data were available. The total sample comprised 22,629 prepacked foods (8557 for the year 2015 and 14,072 for the year 2017), of which 14,154 were in food categories with at least one PHO-containing item (5545 and 8609 for the years 2015 and 2017, respectively; See Supplementary Table S1) The 2017 sample also included all the available pre-packed unprocessed foods such as flour, sugar, and vinegar, as well as food supplements; however, there were no changes in the inclusion criteria for the categories, included to analyses in this study as compared to 2015 (food categories containing PHO-containing foods).

### 2.2. Data Analysis

The data were processed and evaluated using the computer programs Microsoft SQL Server Management Studio V13.0, Microsoft Analysis Services Client Tools 13.0, Microsoft Data Access Components (MDAC) 10.0, Microsoft Excel 2016 (Redmond, WA, USA), and the program tool CLAS V1.0 (Composition and Labelling Information System; Nutrition Institute, Ljubljana, Slovenia). For the purpose of the analyses, the product ingredients were exported from the CLAS database in the form of Microsoft Excel spreadsheets.

The extent of PHO usage in the Slovenian food supply was calculated as a proportion of PHO-containing items in the whole sample of items for each selected food (sub)category, separately for each year. Using the sales data provided by the retailers, the sales-weighted approach was also applied as described previously [29,30,31]. Sales-weighted proportions of PHO-containing items (%) were calculated on the food category level and presented as a proportion of all items available in a given food category. Since several of the same food products were available in different stores, the sales data provided by the retailers were first combined to calculate the number of products sold per year for each food product in the database. Using data on the content of food per package, we calculated the amounts of products sold per year in kg/L (for all available products within the food category and for all available products that contained PHOs). The proportion of PHO-containing prepacked food items (PP) as well as the sale-weighted proportion of PHO-containing foods (sw-PP) in specific food categories are therefore presented as relative values (%). Because the analyses were performed on the whole sample of foods for each food category, the calculated proportions represent an exact value and therefore no confidence intervals are provided.

## 3. Results

**Figure 2:**
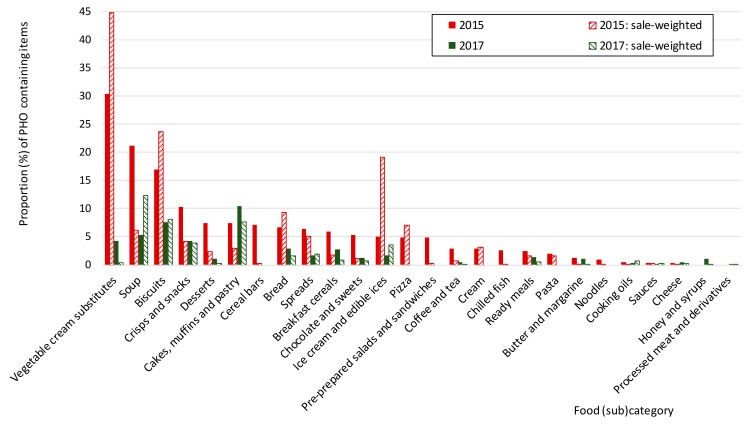
The proportion of PHO-containing prepacked food in the selected food (sub) categories in Slovenia and sales-weighted proportions in 2015 and 2017. Note: Details provided in Supplementary Table S1.

Within the sample of foods collected in 2015, the highest percentage of items with PHOs was found among vegetable cream substitutes (30%) (Figure 2, Supplementary Table S1). The sales-weighted proportion of PHO-containing vegetable cream substitutes was even higher than the availability of those items on the market, as they represented about 45% of all sales. Vegetable cream substitutes were followed by Soups and Biscuits, with 21% and 17% PHO-containing items, respectively. While the amount of sold PHO-containing soups was lower than their actual availability (6%), we observed the opposite trend in prepacked biscuits (sw-PP: 24%), in which PHOs were also used in market-leading brands. Moreover, PHOs were present in 5–10% of items in the following categories: crisps and snacks; desserts; cakes, muffins, and pastries; cereal bars; bread; spreads; breakfast cereals; chocolate and sweets; ice cream and edible ices. Their sales-weighted proportions were very variable, with the exception of ice-creams and edible ices, in which the sales-weighted proportion of PHO-containing items was far above their availability (PP: 5%; sw-PP: 19%).

Notably, a lower proportion of PHO-containing items was observed in the dataset collected in year 2017. Improvements were observed in most of the categories with the highest PP ratios in 2015. In vegetable cream substitutes, the percentage of PHO containing items dropped from 30 down to 4%, in soups it decreased from 21 to 5%, in biscuits from 17 to 8% and in crisps and snacks from 10 to 4%. Improvements were also noted in the following categories: Cereal bars (from 7 down to 0%), desserts (from 7 to 1%), spreads (from 6 to 2%), coffee and tea (from 3 to 0.5%), and chocolate and sweets (from 5 to 1%). However, an increased proportion of PHO-containing items was observed in few of the remaining food categories, particularly in cakes, muffins and pastries (from 7 to 10%), and in honey and syrups (from 0 to 1%).

## 4. Discussion

The reported results show a considerable reduction in the proportion of PHO-containing prepacked foods in most food categories. It is likely that this occurred due to intensified communication of the negative health effects of TFAs on human health since 2014, both within the food industry and in the media. However, PHOs are still used in a notable proportion of foods in some categories—particularly in cakes, muffins and pastries, and in biscuits. While Western European countries have almost completely eliminated TFAs from the analysed food categories, some Central Eastern and South Eastern European countries have seen an increase in the amount of industrially produced TFAs [23,32]. Highly concerning results were published by Stender and colleagues [23]. They focused on prepacked biscuits/cakes/wafers, where the highest TFA levels were expected, due to the common use of PHOs in these foodstuffs. They sampled foods that both contained more than 15 g of fats (per 100 g of food) and had PHOs disclosed at the beginning of the ingredients list. The study was conducted in Slovenia and another five countries in the region. While only 266 samples met the inclusion criteria in the year 2012, 643 foods were sampled in 2014. An increasing trend in the availability of biscuits/cakes/wafers with over 2% of TFA was observed in all investigated countries. A comparison of the situation between 2012 and 2014 showed that—looking at the entire sample—the number of available items with more than 2% TFA almost doubled from an average of 33 to an average of 68 products per country, while in Slovenia this increase was even more evident (294%). The average content of TFAs in the Slovenian sample also increased, from 15.5% to 17.9% [23].

The strength of the study by Stender et al. [23] was its systematic sampling methodology and the fact that all sampled foods were subjected to chemical determination of TFAs. However, the limitations were that only PHO-containing foods were sampled, and that the study only focused on biscuits/cakes/wafers. Therefore, the study did not provide details on the proportion of PHO/TFA containing foods on the entire market. The approach used in our study (which represents the first phase of the TFFS project) was very different. We decided to systematically screen all prepacked foods across various food categories without excluding non-PHO containing items, which resulted in an extremely large sample size. Thus, the burden of performing chemical analyses on all the sampled products would have been extreme, and not a rational option. Therefore, we considered the presence of any PHO content in food as a relevant risk that such food also contains a notable amount of TFAs. Such an approach is also in line with the decision of the Food and Drug Administration (FDA), that PHOs are no longer generally recognized as safe [18].

In our 2015 dataset, numerous PHO-containing foods were found among biscuits, which is similar to the observation of Stender et al. [18] a year earlier. However, while they observed that the availability of foods with a notable amount of TFA (>2%) among biscuits/cakes/wafers increased considerably from 2012 to 2014, this trend seems to have changed since 2015. The proportion of PHO-containing items among biscuits, desserts, and cakes, muffins and pastries plummeted by 46% (from 13 to 7%) from 2015 to 2017. Of these, a most notable improvement was detected amongst biscuits and desserts (which reduced by 55% and 86%, respectively), while an increase was observed in cakes, muffins and pastries (by 42%). Similar trends can be observed when the proportions were adjusted with sales data: a 61% reduction was observed for the combined sample of all three categories, with a notable reduction in biscuits and desserts (66 and 89%, respectively), but a striking 167% increase in cakes, muffins and pastries.

In the data collected for 2015, these categories were not identified as the categories with the highest proportions of PHO-containing foods. The highest proportions were found in vegetable cream substitutes (30%)—an alternative for milk-based whipping and cooking cream, followed by soups (21%). However, in vegetable cream substitutes, this percentage dropped considerably in 2017, as only one such product with a low market share was labelled as still containing PHOs. In the case of soups, it should be noted that due to a low content of total fat PHO content in soups is not expected to be an important source of TFAs. We also noted drastic improvements in two other food categories with substantial penetration of PHO-containing items in 2015; namely, cereal bars (complete elimination) and crisps and snacks, where we observed a reduction of such items by 59%. However, this reduction was much less prominently expressed after adjusting for market share (only for 4%).

The strength of this study is a very robust screening of the food supply, and the use of a sales-weighting approach to adjust the proportion of PHO-containing foods by their market share. Some limitations should also be noted. Firstly, the sample of products was extremely large; therefore, we were not able to use laboratory analyses to determine the actual content of TFA in the foods. Thus, some of the foods that had PHOs on the list of ingredients may actually contain minimal amounts of TFAs. For example, the amount of the TFAs in foods depends on the amount of PHO added, the type of hydrogenation made, and the final fat content of the food. In the next step of the TFFS project, the results of this study will be used to select representative samples of foods for chemical analyses (ongoing study, focusing on following food categories: vegetable cream substitutes; soup; biscuits; crisps and snacks; desserts; cakes, muffins and pastry; bread; spreads; breakfast cereals; chocolate and sweets; ice cream and edible ices. Additionally, margarines were also selected for sampling, as these were a major source of TFAs in the past [33]). Secondly, while the data were collected from major retailers within the nation-wide store networks, we are aware that we did not cover the entire Slovenian market. However, the retailers included in this study accounted for over 50% of the total Slovenian market share and are, as such, a good estimation of the whole market. Lastly, the study did not examine non-prepacked food items sold in supermarkets and bakeries, where no labelling information is provided. Nevertheless, non-prepacked foods are subject to other phases of the TFSS project, and have been included as a part of the sampling for laboratory analyses. We should also note that although we did not observe that the content of PHOs in foods would be a factor in lowering consumer preferences towards such products, this study was not designed to measure this. Consumer purchasing decisions depend on several different factors, including brand name, price, and quality of the product. It has been reported in other countries that PHOs are generally not perceived as unhealthy [21], but this question has not yet been investigated among Slovenian population.

## 5. Conclusions

The elimination of industrially produced TFAs from the food supply has been described as an easily achievable dietary intervention for preventing non-communicable disease, of which CVDs are the most common [34]. Regulation of the mandatory labelling of TFAs/PHOs and the voluntarily reduction of PHO usage have both been previously shown to yield limited success [35], which was also shown in our study. Despite major efforts to communicate the risks related to TFA consumption with different stakeholders and the public, and the fact that, in some jurisdictions, PHOs were specifically assigned as an unsafe food ingredient, PHOs can still be found in a notable proportion of foods in the Slovenian food supply, particularly in cakes, muffins, pastries, and biscuits. Legislative restriction of the use of industrially produced TFAs in foods would assure further improvement in protecting public health. While our results show that in Slovenia the use of PHOs is now limited to only a few food categories, we cannot exclude the fact that such foods still present a relevant health risk for specific populations, which are consuming high quantities of such foods.

## Supplementary Materials

The following are available online at www.mdpi.com/2072-6643/10/3/355/s1, Table S1: The proportion of PHO-containing pre-packed foods in the selected food (sub)categories in Slovenia.
