# Supplementary material for: Limiting trans Fats in Foods: Use of Partially Hydrogenated Vegetable Oils in Prepacked Foods in Slovenia
Source: Nutrients. 2018 Mar 15;10(3):355. doi: 10.3390/nu10030355 (PMC5872773; doi:10.3390/nu10030355)
Supplement: Supplementary file 1 [file nutrients-10-00355-s001.pdf]

Supplementary Table S1. The proportion of PHO-containing pre-packed foods in the selected food (sub)categories in Slovenia

	2015			2017		
**Food (sub)category**	**Number of all food samples**	**Proportion of PHO containing items (%)**	**Sales-weighted proportion of PHO-containing items (%)**	**Number of all food samples**	**Proportion of PHO containing items (%)**	**Sales-weighted proportion of PHO-containing items (%)**
Vegetable cream substitutes	23	30,4	44,8	24	4,2	0,4
Soup	151	21,2	6,2	207	5,3	12,3
Biscuits	651	16,9	23,6	941	7,5	8,0
Crisps and snacks	205	10,2	4,1	308	4,2	3,9
Desserts	148	7,4	2,4	194	1,0	0,3
Cakes, muffins and pastry	286	7,3	2,9	423	10,4	7,7
Cereal bars	56	7,1	0,2	48	0,0	0,0
Bread	120	6,7	9,3	141	2,8	1,7
Spreads	235	6,4	5,0	416	1,7	1,9
Breakfast cereals	256	5,9	1,7	333	2,7	0,9
Chocolate and sweets	806	5,3	1,1	1308	1,2	0,6
Ice cream and edible ices	179	5,0	19,1	244	1,6	3,6
Pizza	21	4,8	7,0	26	0,0	0,0
Pre-prepared salads and sandwiches	21	4,8	0,3	10	0,0	0,0
Coffee and tea	457	2,8	0,7	790	0,5	0,1
Cream	71	2,8	3,0	81	0,0	0,0
Chilled fish	38	2,6	0,0	29	0,0	0,0
Ready meals	208	2,4	1,5	228	1,3	0,5
Pasta	296	2,0	1,5	494	0,0	0,0
Butter and margarine	84	1,2	0,1	93	1,1	0,1
Noodles	105	1,0	0,0	123	0,0	0,0
Cooking oils	190	0,5	0,1	321	0,3	0,7
Sauces	276	0,4	0,2	576	0,2	0,2
Cheese	292	0,3	0,1	467	0,4	0,2
Honey and syrups	6	0,0	0,0	203	1,0	0,0
Processed meat and derivatives	364	0,0	0,0	581	0,2	0,1
